# Dal81 Regulates Expression of Arginine Metabolism Genes in *Candida parapsilosis*

**DOI:** 10.1128/mSphere.00028-18

**Published:** 2018-03-07

**Authors:** Siobhan A. Turner, Qinxi Ma, Mihaela Ola, Kontxi Martinez de San Vicente, Geraldine Butler

**Affiliations:** a School of Biomolecular and Biomedical Science, Conway Institute, University College Dublin, Belfield, Dublin, Ireland; Carnegie Mellon University

**Keywords:** *Candida*, nitrogen metabolism, opportunistic fungi

## Abstract

Fungi can use a wide variety of nitrogen sources. In the absence of preferred sources such as ammonium, glutamate, and glutamine, secondary sources, including most other amino acids, are used. Expression of the nitrogen utilization pathways is very strongly controlled at the transcriptional level. Here, we investigated the regulation of nitrogen utilization in the pathogenic yeast *Candida parapsilosis*. We found that the functions of many regulators are conserved with respect to *Saccharomyces cerevisiae* and other fungi. For example, the core GATA activators *GAT1* and *GLN3* have a conserved role in nitrogen catabolite repression (NCR). There is one ortholog of *GZF3* and *DAL80*, which represses expression of genes in preferred nitrogen sources. The regulators *PUT3* and *UGA3* are required for metabolism of proline and γ-aminobutyric acid (GABA), respectively. However, the role of the Dal81 transcription factor is distinctly different. In *S. cerevisiae*, Dal81 is a positive regulator of acquisition of nitrogen from GABA, allantoin, urea, and leucine, and it is required for maximal induction of expression of the relevant pathway genes. In *C. parapsilosis*, induction of GABA genes is independent of Dal81, and deleting *DAL81* has no effect on acquisition of nitrogen from GABA or allantoin. Instead, Dal81 represses arginine synthesis during growth under preferred nitrogen conditions.

**IMPORTANCE** Utilization of nitrogen by fungi is controlled by nitrogen catabolite repression (NCR). Expression of many genes is switched off during growth on nonpreferred nitrogen sources. Gene expression is regulated through a combination of activation and repression. Nitrogen regulation has been studied best in the model yeast *Saccharomyces cerevisiae*. We found that although many nitrogen regulators have a conserved function in *Saccharomyces* species, some do not. The Dal81 transcriptional regulator has distinctly different functions in *S. cerevisiae* and *C. parapsilosis*. In the former, it regulates utilization of nitrogen from GABA and allantoin, whereas in the latter, it regulates expression of arginine synthesis genes. Our findings make an important contribution to our understanding of nitrogen regulation in a human-pathogenic fungus.

## Abstract (precis)

Utilization of nitrogen by fungi is controlled by nitrogen catabolite repression (NCR). Expression of many genes is switched off during growth on nonpreferred nitrogen sources. Gene expression is regulated through a combination of activation and repression. Nitrogen regulation has been studied best in the model yeast *Saccharomyces cerevisiae*. We found that although many nitrogen regulators have a conserved function in *Saccharomyces* species, some do not. The Dal81 transcriptional regulator has distinctly different functions in *S. cerevisiae* and *C. parapsilosis*. In the former, it regulates utilization of nitrogen from GABA and allantoin, whereas in the latter, it regulates expression of arginine synthesis genes. Our findings make an important contribution to our understanding of nitrogen regulation in a human-pathogenic fungus.

## INTRODUCTION

Nitrogen is a key component of all proteins, and fungi can use a variety of compounds as a sole source. Preferred nitrogen sources include glutamate, glutamine, ammonium, and peptones (1). When these sources are available, expression of genes associated with the utilization of poor nitrogen sources is repressed, in a process called nitrogen catabolite repression (NCR) (2–5). Regulation of nitrogen metabolism has been particularly well characterized in *Saccharomyces cerevisiae*, where expression of many nitrogen-responsive genes is controlled by four GATA-type transcription factors (2). Two (Gzf3 and Dal80) are repressors that switch off expression of target genes when preferred nitrogen sources are available (6, 7). The other two (Gat1 and Gln3) are activators and are required for expression of genes involved in acquisition of nitrogen from poor sources. During growth on preferred nitrogen sources, Gat1 and Gln3 are phosphorylated and are sequestered in the cytoplasm by the activity of Ure2 (8–11). When only poor nitrogen sources are present, Gat1p and Gln3p are dephosphorylated and translocated to the nucleus, where they drive expression of genes involved in assimilating and catalyzing degradation of nonpreferred nitrogen sources (1, 12–14). Nitrogen-rich cellular processes, such as protein translation and amino acid biosynthesis, are downregulated during nitrogen starvation (2). Many proteins, including membrane transporters that import ammonium (15, 16), amino acid transporters (17), and secreted proteases that degrade environmental proteins to release amino acids (4), are subject to NCR at both the mRNA and protein levels.

Several other proteins regulate expression of nitrogen metabolism genes. For example, Gcn4 regulates expression of many amino acid biosynthesis genes (18). In *S. cerevisiae*, Gcn4 is strongly regulated at the translational level through competition with small upstream open reading frames (uORFs) (19), which are conserved in *Candida albicans*, though they may not function in exactly the same manner (20, 21). In *S. cerevisiae*, translation of Gcn4 is increased during amino acid starvation, leading to derepression of most amino biosynthetic pathways (22). Growth on some amino acids as the sole nitrogen source also induces expression of specific transcription factors that are required for utilization of these sources. For example, growth of *S. cerevisiae* on proline induces expression of Put3, which drives expression of genes involved in proline import and utilization (23, 24), and induction of arginine utilization genes requires Arg80 and Arg81 (25, 26). In *S. cerevisiae*, Dal81 is required for the expression of genes involved in the metabolism of allantoin, γ-aminobutyric acid (GABA), leucine, and urea (27, 28). During growth on GABA, Dal81 acts with Uga3 to drive expression of GABA-responsive genes, including the *UGA* regulon (29). When allantoin is the sole nitrogen source, Dal81, together with Dal82, controls expression of the *DUR* and *DAL* genes (30). In addition, Dal81 acts with Stp1 to regulate expression of amino acid permease genes, especially those that import leucine (31).

NCR has been characterized in several other fungi, including *Yarrowia lipolytica*, *Aspergillus nidulans*, *Neurospora crassa*, *Cryptococcus neoformans*, and *Candida albicans* (3, 32–34). The roles of the GATA activators are generally well conserved. For example, in *C. albicans*, Gat1 and Gln3 are required for derepression of expression of the ammonium permease *MEP2* and the secreted aspartyl protease *SAP2* as well as the urea, allantoin, and GABA metabolism genes *DUR1*, *DUR2*, *DAL5*, and *UGA4* during growth on preferred nitrogen sources (34–38). Loss of either *GAT1* or *GLN3* leads to attenuated virulence of *C. albicans* in a mouse model of infection (34, 36). Deleting *GAT1* or *GLN3* also reduces formation of chlamydospores, unusual globular structures that are formed during growth on some media (39).

The GATA repressors are not as well characterized in other fungi as they are in *S. cerevisiae*. *DAL80* and *GZF3* are ohnologs resulting from whole-genome duplication (WGD) in the ancestor of *S. cerevisiae* (40, 41). Fungi that diverged from *S. cerevisiae* before the WGD therefore have only one ortholog. The role of the single *C. albicans* ortholog (called *GZF3*) has not been studied. *Y. lipolytica* encodes four GATA transcription factors, including two (Gzf1 and Gzf2) that are similar to Gat1 and Gln3; Gzf3, which is related to *S. cerevisiae* Gzf3 and Dal80; and Gzf4, which is more closely related to iron response regulators (32). Gzf3 represses expression of nitrogen utilization genes in *Y. lipolytica*, similarly to *S. cerevisiae*, whereas Gzf4 is more closely related to iron response regulators (32). In many filamentous fungi, the GATA transcription factor AreA (Gat1 family) is an activator of gene expression whereas AreB (Gzf3) represses expression (42, 43).

Here, we used reverse genetics to assess the role of NCR regulators in the pathogenic yeast *Candida parapsilosis*, a relative of *C. albicans* in the CUG-Ser clade (44, 45). We found that *GAT1* and *GLN3* orthologs are activators of NCR and that Gzf3 acts as a general repressor of nitrogen catabolism genes. The roles of Put3 and Uga3 as regulators of proline and GABA metabolism, respectively, are also conserved with *S. cerevisiae*. However, the role of Dal81 is different. *DAL81* is not required for allantoin or GABA metabolism in either *C. albicans* or *C. parapsilosis*. Transcriptomic analysis shows that in *C. parapsilosis*, Dal81 acts as a regulator of genes required for metabolism of arginine and especially as a repressor of arginine biosynthesis and transport. There has therefore been substantial rewiring of the Dal81 transcriptional network between *S. cerevisiae* and *C. parapsilosis*.

## RESULTS

### Nitrogen catabolite repression in *C. parapsilosis*.

Fungal species preferentially use nitrogen sources such as glutamate, glutamine, ammonium, and peptones, but they can also utilize nonpreferred sources, including allantoin, γ-aminobutyric acid (GABA), and other amino acids. We first tested if gene expression is subject to nitrogen catabolite repression in *C. parapsilosis* CLIB214 in a manner similar to that seen with other fungi by comparing the levels of gene expression of wild-type cells growing in media containing a preferred nitrogen source (yeast nitrogen base [YNB] with glucose and ammonium sulfate) and in media with a nonpreferred source (YNB with glucose and isoleucine).

Overall, 731 genes were upregulated and 292 genes were downregulated in cells grown on isoleucine compared to ammonium sulfate as the sole nitrogen source (see Data Set S1 in the supplemental material). Upregulated genes were enriched in transmembrane transporters and permeases, including transporters of ammonium (*MEP1* and *MEP2*), urea (*DUR3* and *DUR4*), allantoate (*DAL4*, *DAL7*, and *DAL9*), and oligopeptides (*OPT2*, *OPT5*, *OPT6*, and *OPT8*). Genes involved in amino acid transport and synthesis, especially those involved in arginine biosynthesis (e.g., *ARG1*, *ARG3*, *ARG5,6* and *CPA2*), were also upregulated. Expression of the putative NCR activator *GAT1* was increased by a log_2_ fold change (log_2_FC) value of ~6 and that of the putative NCR repressor *GZF3* by a log_2_FC of ~2. These responses are consistent with an increase in levels of nitrogen scavenging pathways during growth on nonpreferred nitrogen sources and are similar to results of previous analyses of derepression of pathways subject to NCR in *S. cerevisiae* (4, 46).

10.1128/mSphere.00028-18.6 DATA SET S1  RNA-seq analysis of nitrogen response of *C. parapsilosis* wild-type and *dal81* deletion strains. Download DATA SET S1, XLS file, 1.3 MB. Copyright © 2018 Turner et al. 2018 Turner et al. This content is distributed under the terms of the Creative Commons Attribution 4.0 International license.

### Identification of NCR regulators in *C. parapsilosis*.

**FIG 1:**
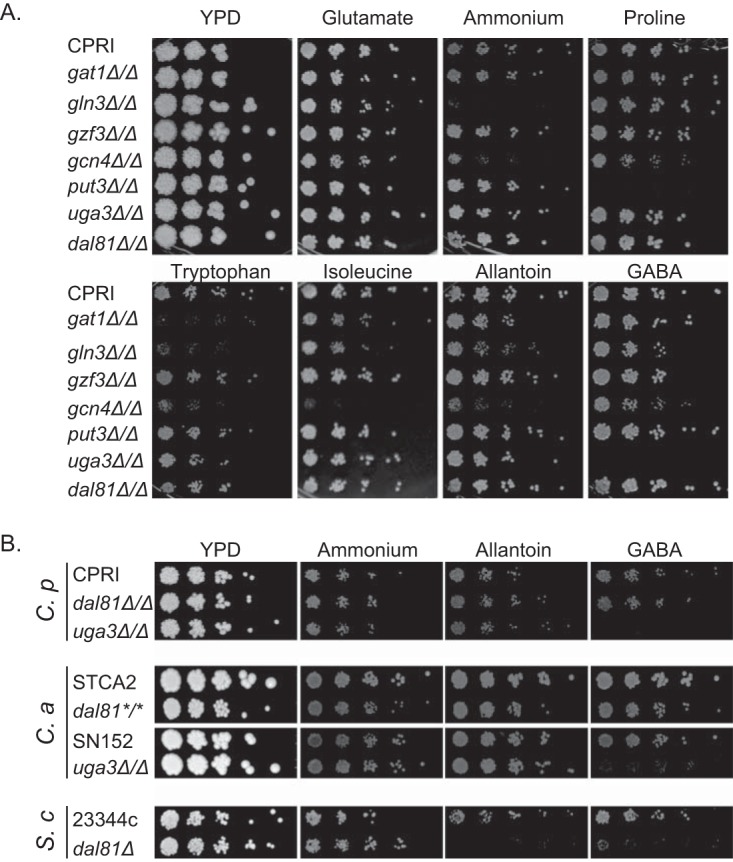
Identification of regulators of nitrogen metabolism in *Candida* species. (A) *C. parapsilosis* strains were grown overnight in YPD, and increasing 1/5 dilutions were pinned on YPD and on minimal media (YNB with glucose and without amino acids or ammonium sulfate) supplemented with a 10 mM concentration of the indicated amino acid or allantoin or with 0.5% ammonium sulfate. Plates were photographed after 72 h, and growth of the deletion strains was compared to the growth of the control *C. parapsilosis* CPRI strain. (B) Disrupting *DAL81* reduced growth of *S. cerevisiae* when allantoin or GABA was the sole nitrogen source but had no effect on growth of *C. albicans* and *C. parapsilosis*. Deleting *UGA3* reduced growth of both *C. parapsilosis* and *C. albicans* on GABA. *C. p*, *C. parapsilosis*; *C. a*, *C. albicans*, *S. c*, *S. cerevisiae*. The relevant control strains for each species were used (*C. parapsilosis* CPRI, *C. albicans* STCA2 [for *dal81*], *C. albicans* SN152 [for *uga3Δ* {49}], and *S. cerevisiae* 23344c). The asterisks indicate that *DAL81* was edited in *C. albicans* by introducing two stop codons. For the *S. cerevisiae* strains, uracil was added to the media at a final concentration of 0.08 g/liter. For *C. albicans* SN152 and the corresponding *uga3*Δ strain, arginine was added to reach a final concentration of 0.05 g/liter.

The roles of *C. parapsilosis* orthologs of known nitrogen regulators in *S. cerevisiae* were next tested by characterizing the phenotype of gene deletion strains growing on various nitrogen sources (Fig. 1). *GAT1* and *GZF3* deletions in *C. parapsilosis* were previously described by Holland et al. (47); deletions of *GLN3*, *PUT3*, *GCN4*, *UGA3*, and *DAL81* were constructed using a similar methodology. Most of the deletion strains grew well on rich media with complex nitrogen sources (yeast extract-peptone-dextrose [YPD], glucose with yeast extract and peptone) and on YNB plus glucose when glutamate (a preferred nitrogen) was used as the sole nitrogen source. Loss of *GLN3* resulted in poor growth on ammonium sulfate and under most of the nitrogen-limiting conditions tested, similarly to the orthologous deletion in *C. albicans* (36). Deletion of the *GAT1* activator led to dramatically reduced growth on tryptophan, which is also observed in *C. albicans* (36). Deleting *GAT1* resulted in a minor growth defect on glutamate, the preferred nitrogen source, which has not been reported in other fungal species. Overall, the growth phenotypes support the hypothesis that Gat1 and Gln3 are activators of genes required for utilization of nonpreferred nitrogen sources in *C. parapsilosis*, similarly to *S. cerevisiae* and *C. albicans*.

There is one ortholog of *S. cerevisiae GZF3* and *DAL80*, called *GZF3*, in *Candida* species (40, 41). Deleting *GZF3* in *C. parapsilosis* had no effect on growth on any of the nitrogen sources tested (Fig. 1). In *S. cerevisiae*, Gzf3 and Dal80 repress expression of metabolic genes such as those encoding transporters of ammonium and amino acids during growth on preferred sources (6). *GAT1* is also one of the targets (48). We therefore tested the effect of deleting *C. parapsilosis GZF3* on expression of some potential targets, including *MEP2* (ammonium permease and sensor of nitrogen starvation), *GAP2* (amino acid permease), and *GAT1*, which were upregulated when *C. parapsilosis* was grown on isoleucine as the major nitrogen source (Data Set S1). Table 1 shows that expression of all three genes was increased (8-fold to 80-fold) when *GZF3* was deleted during growth on rich media with complex sources of nitrogen (YPD). Gzf3 therefore represses expression of genes required for metabolism of nonpreferred nitrogen sources.

**TABLE 1:** Relative expression levels of NCR-sensitive genes in *gzf3Δ* strains grown in YPD

Gene	Fold change (2^−ΔΔ*CT*^)^a^		*P* value^b^
	*C. parapsilosis* CPRI	*C. parapsilosis gzf3Δ/Δ*	
*GAP2*	1.0 (0.32–3.15)	8.85 (2.54–30.87)	0.0006
*MEP2*	1.0 (0.47–2.13)	79.5 (27.7–228.2)	0.0000001
*GAT1*	1.0 (0.41–2.46)	23.43 (7.0–78.43)	0.003

a Threshold cycle (*C*_*T*_) values were normalized to *ACT1*, and the expression level in *C. parapsilosis* CPRI was set to 1. Gene expression ranges (standard deviations) are shown in parentheses.

b *P* values comparing expression levels in *C. parapsilosis* CPRI and *gzf3Δ* strains were calculated from three biological replicates by the use of a two-tailed Student *t* test.

The roles of some other transcription factor orthologs are also conserved in *S. cerevisiae* and *C. parapsilosis*. Deleting *GCN4* in *C. parapsilosis* reduced growth on most media (Fig. 1). This is similar to results seen with both *S. cerevisiae* and *C. albicans*, where Gcn4 plays a key role in coordinating the response to amino acid starvation (18, 21). *PUT3* was required for utilization of proline only and *UGA3* for utilization of GABA only (Fig. 1). However, deleting *DAL81* had no effect on growth under any set of conditions. To test whether this phenotype is unique to *C. parapsilosis*, we edited the *DAL81* ortholog in *C. albicans* by introducing two stop codons by the use of a clustered regularly interspaced short palindromic repeat (CRISPR)-Cas9-based method. We found that the edited strain had no defect in growth on various nitrogen sources, even when allantoin or GABA was the sole nitrogen source (Fig. 1). In *S. cerevisiae*, Dal81 is required for metabolism of GABA (together with Uga3) and of allantoin (together with Dal82) (Fig. 1) (28, 29). The role of Uga3 in GABA metabolism is conserved in both *C. albicans* (49) and *C. parapsilosis* (Fig. 1B). There is no ortholog of *DAL82* in *Candida* species.

### Role of *C. parapsilosis* Dal81.

**FIG 2:**
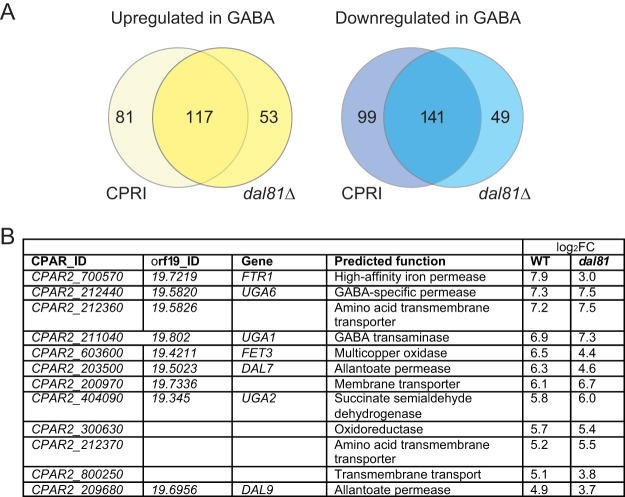
GABA induction of gene expression does not require *DAL81*. (A) Genes upregulated and downregulated in *C. parapsilosis* CPRI and *dal81*Δ strains grown in GABA as a sole nitrogen source. Gene expression is compared to growth on ammonium sulfate for each strain. (The full list is available in Data Set S1.) (B) Genes with the highest induction in expression under conditions of growth on GABA. The log_2_ fold change in *C. parapsilosis* CPRI and *dal81*Δ strains is shown. (The full list is available in Data Set S1.) WT, wild type.

Our observation that deleting *DAL81* did not affect growth of either *C. albicans* or *C. parapsilosis* on allantoin or GABA suggests that the protein has functions in *Candida* that are different from its functions in *Saccharomyces* species. To determine if *DAL81* plays any role in regulating expression of genes required for GABA metabolism in *C. parapsilosis*, we compared the gene expression profiles of wild-type and *dal81* deletion strains growing in minimal media supplemented with either ammonium sulfate or GABA as the sole nitrogen source. We used *C. parapsilosis* CPRI as a control strain (47). The gene deletions were constructed by replacing one allele with *HIS1* from *C. dubliniensis* and one with *LEU2* from *C. maltosa*. *C. parapsilosis* CPRI also contains one *C. dubliniensis HIS1* (*CdHIS1*) allele and one *C. maltosa LEU2* (*CmLEU2*) allele (47). In *C. parapsilosis* CPRI, expression of 198 genes was increased by a log_2_FC of >1 during growth on GABA compared to ammonium sulfate, and expression of 117 of these genes was also increased even when *DAL81* is deleted (Fig. 2). Similarly, expression of 240 genes was decreased in the control strain during growth on GABA relative to ammonium sulfate, and 141 of these were also downregulated in the *DAL81* deletion. Growth on GABA resulted in increased expression of genes involved in GABA metabolism, including the UGA transaminase (*UGA1*) and succinate-semialdehyde dehydrogenase (*UGA2*), even when *DAL81* was deleted, by a log_2_FC of >5 (Data Set S1). We also used quantitative real-time PCR (RT-PCR) to confirm that *DAL81* is not required for the GABA-specific induction of metabolic genes (Table 2). Expression of *UGA1* and *UGA2* was induced >100-fold during growth in GABA, in the presence and absence of *DAL81*.

**TABLE 2:** GABA induction of gene expression in control and *dal81Δ* strains of *C. parapsilosis*

Gene	Fold change (2^−ΔΔ*CT*^)^a^				*P* value^b^
	*C. parapsilosis* CPRI		*C. parapsilosis dal81*Δ/Δ		
	Ammonium	GABA	Ammonium	GABA	
*UGA1*	1.0 (0.2–4.7)	142.5 (45.4–447.1)	1.0 (0.2–6.0)	118.6 (54.8–256.9)	0.57
*UGA2*	1.0 (0.2–6.1)	219.3 (31.7–1,516.6)	1.0 (0.2–6.8)	113.1 (28.7–445.3)	0.95

a *C*_*T*_ values were normalized to *ACT1* to generate Δ*C*_*T*_ values for each gene, and the expression level during growth in ammonium sulfate was set to 1. Gene expression ranges (standard deviations) are shown in parentheses. Cells were grown in YNB/glucose with 0.5% ammonium sulfate or 10 mM GABA.

b *P* values comparing expression levels of control (CPRI) and *dal81* deletion strains in GABA were calculated from three biological replicates by the use of a two-tailed Student *t* test.

In *S. cerevisiae*, GABA is imported by the Uga4 permease (50). There is no syntenic homolog of *UGA4* in *C. parapsilosis* (51, 52). GABA may be imported by *UGA6* (*CPAR2_212440*) and/or *CPAR2_212360*, a member of the same permease family, because expression of these genes was increased by a log_2_FC of >7.0 in media containing GABA (Fig. 2; see also Data Set S1). However, expression of both was also induced when isoleucine is the sole nitrogen source. Expression of *DAL9*, encoding a putative allantoate permease, was induced by GABA in the presence or absence of *DAL81* (Fig. 2). It is therefore likely that in *C. parapsilosis*, and possibly in other *Candida* species, *DAL81* does not regulate expression of GABA or allantoate metabolism genes, as indicated by the lack of a growth defect (Fig. 1).

Deleting *DAL81* had little effect on overall gene expression levels when cells were grown in ammonium sulfate. Expression of only 40 genes was decreased in the *dal81* deletion strain relative to *C. parapsilosis* CPRI, and expression of 15 genes was increased with a log_2_FC of >1 (Data Set S1). However, expression of arginine biosynthesis genes *CPA2*, *ARG4*, and *ARG5*,*6* was increased and expression of *CAR1* and *CAR2*, involved in arginine degradation, was decreased (Data Set S1). Expression of *DUR1*,*2* (urea amidolyase) and of *DUR3* (a putative urea transporter) was reduced (by a log_2_FC value greater than 1.8) in the *dal81* deletion strain compared to a control strain grown in ammonium sulfate, suggesting that its role in regulation of urea metabolism may be partially conserved with *S. cerevisiae* (Data Set S1) (53). The expression of galactose metabolism genes (*GAL1* and *GAL7*) was also reduced. However, expression of the *DUR* genes was induced by growth on GABA, even when *DAL81* was deleted (Data Set S1).

**FIG 3:**
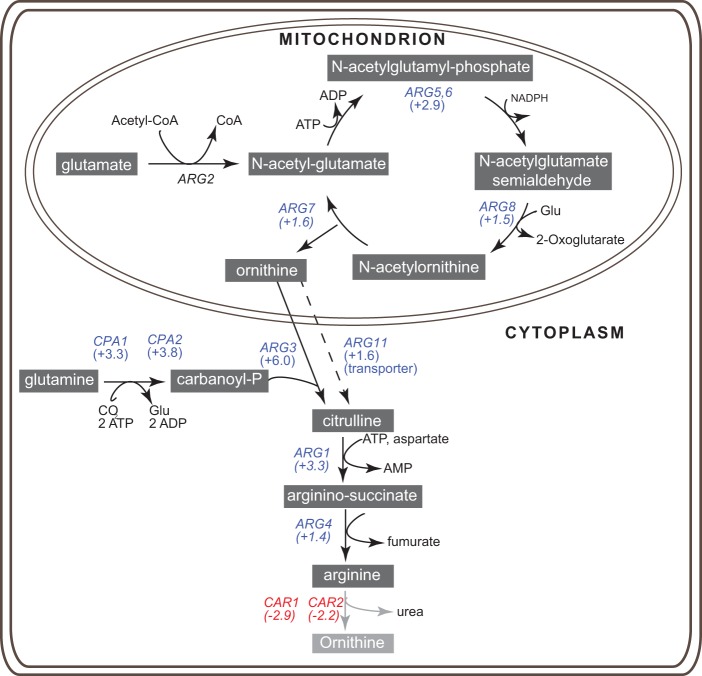
*DAL81* regulates expression of arginine metabolism in *C. parapsilosis*. The diagram shows a schematic representation of the arginine biosynthetic pathway in yeast. Expression of the genes highlighted in blue was increased in a *dal81* deletion strain of *C. parapsilosis* growing in YPD. Expression of genes shown in red was decreased. The gene shown in black (*ARG2*) had no detectable change in expression. The log_2_FC data are indicated in parentheses. (Full data are available in Data Set S1.) CoA, coenzyme A.

To further characterize the role of *DAL81*, we compared the gene expression profile of a *dal81* deletion strain to that of the wild type under conditions of cell growth in YPD, a rich medium containing complex nitrogen sources, including yeast extract and peptones. Expression of 493 genes was increased and of 337 genes was decreased in the *dal81* deletion strain (Data Set S1). The upregulated genes were involved in processes associated with ribosome biogenesis and, in particular, with arginine metabolism (Data Set S1). Expression of several genes required for arginine biosynthesis and transport was increased, whereas expression of arginine catabolic genes was decreased (Fig. 3).

To confirm that the observed increase in expression of *ARG* genes was caused by deleting *dal81* and was not a result of a secondary mutation, we restored an intact copy of *DAL81* at one of the deleted loci in *C. parapsilosis* (Fig. S1). Expression of *ARG1* and *ARG3* was found to have increased 6- to 60-fold when *DAL81* was deleted and was restored to wild-type levels in the complemented strain (Table 3). We also used CRISPR-based editing to delete *DAL81* in a clinical isolate, *C. parapsilosis* 90-137 (54). Expression of *ARG3* was increased >200-fold in the *dal81* deletion strain (Table 3). In addition, deleting *DAL81* did not reduce growth of *C. parapsilosis* 90-137 on GABA or allantoin as the sole nitrogen source (Fig. S2). The effect of deletion of *DAL81* on expression of arginine genes and the lack of a role in regulating allantoin and GABA metabolism are therefore not restricted to one isolate of *C. parapsilosis*. Deleting *DAL81* did not reduce growth of *C. parapsilosis* or *C. albicans* on arginine or ornithine as the sole nitrogen source and did not affect susceptibility to canavanine, which is a toxic analog of arginine (Fig. S3). It is, however, likely that expression of arginine biosynthesis genes in *Candida* species is also regulated by other activators, similarly to *S. cerevisiae* (25, 26).

10.1128/mSphere.00028-18.1 FIG S1  Complementation of *C. parapsilosis dal81* deletion. (A) *DAL81* was deleted by replacing both alleles with the auxotrophic markers *CmLEU2* and *CdHIS1* (47). (B) To reintroduce the wild-type gene, the *DAL81* ORF was cloned into plasmid pSFS2A (80). The selectable marker (*SAT1* cassette) was flanked on one side by *DAL81*, and the other by part of the *CdHIS1* ORF used to delete one of the *DAL81* alleles. A linear fragment was transformed into the knockout strain. (C) Structure of one of the alleles after reintegration of *DAL81* in the knockout strain. The construct was verified using primers (purple arrows) to amplify inside ORF (ORF presence check) and around the 5′ and the 3′ ends of the integration site. (E) The *DAL81* ORF (802-bp) site was amplified from the wild-type strain and four putative reintegration isolates (RI 1, 2, 3, and 4), but not from the deletion strain (KO [knockout]). A 1,098-bp fragment was amplified from the wild-type strain and RI 1 but not from the others. A 1,103-bp fragment was amplified from the 3′ end of RI 1 only. The fragments from the 5′ and 3′ ends of RI 1 were sequenced to confirm the construction. Download FIG S1, EPS file, 0.9 MB. Copyright © 2018 Turner et al. 2018 Turner et al. This content is distributed under the terms of the Creative Commons Attribution 4.0 International license.

10.1128/mSphere.00028-18.2 FIG S2  Deleting *DAL81 in C. parapsilosis* 90-137 did not affect growth on allantoin or GABA as the sole nitrogen source. Both alleles of *DAL81* were deleted in *C. parapsilosis* 90-137 using CRISPR. Strains were grown overnight in YPD, and increasing 1/5 dilutions were plated on minimal media (YNB with glucose without amino acids or ammonium sulfate) supplemented with 0.5% ammonium sulfate or with 10 mM allantoin or GABA where indicated. Download FIG S2, TIF file, 2.5 MB. Copyright © 2018 Turner et al. 2018 Turner et al. This content is distributed under the terms of the Creative Commons Attribution 4.0 International license.

10.1128/mSphere.00028-18.3 FIG S3  Disrupting *DAL81* did not affect growth in *C. albicans* or *C. parapsilosis* during growth on arginine, ornithine, or canavanine as sole nitrogen source. *C. albicans* and *C. parapsilosis* strains were grown overnight in YPD, and increasing 1/5 dilutions were pinned on minimum media (YNB with glucose and without amino acids or ammonium sulfate), supplemented with a 10 mM concentration of arginine or ornithine or with 0.5% ammonium sulfate. Canavanine was added at a final concentration of 80 µg/ml where indicated. Plates were photographed after 72 h. Growth levels of the mutants were compared to the level seen with the relevant control for each species (*C. albicans* STCA2, *C. parapsilosis* CPRI, or *C. parapsilosis* 90-137). C.a, *C. albicans*; C.p, *C. parapsilosis*; *, edited strains; Δ, gene deletion strains. Download FIG S3, EPS file, 1.1 MB. Copyright © 2018 Turner et al. 2018 Turner et al. This content is distributed under the terms of the Creative Commons Attribution 4.0 International license.

**TABLE 3:** Reintroducing *DAL81* restores expression of *ARG* genes in *C. parapsilosis* cells grown in YPD

Gene	Fold change (2^−ΔΔ*CT*^)^a^				
	CLIB214			90-137	
	WT^b^	*dal81*Δ/Δ	*dal81*Δ::*DAL81*	WT^b^	*dal81*Δ/Δ
*ARG1*	1.0 (0.1–10.1)	6.3 (1.8–22.2)	0.6 (0.3–1.4)		
*ARG3*	1.0 (0.1–12.1)	62.2 (15.3–252.1)	0.7 (0.3–1.4)	1.0 (0.9–1.1)	244.7 (179.6–333.5)

a *C_T_* values were normalized to *ACT1*, and the expression level in *C. parapsilosis* CLIB214 or *C. parapsilosis* 90-137 was set to 1. Gene expression ranges (standard deviations) are shown in parentheses.

b WT, wild type.

## DISCUSSION

The capacity to utilize nonpreferred nitrogen sources is an important virulence factor as it allows growth and survival of pathogens in diverse host niches. For example, deleting the core regulators of nitrogen assimilation, *GAT1* and *GLN3*, reduces virulence in *C. albicans* (34, 36). Here, we show that growth on poor nitrogen sources (e.g., isoleucine) triggers derepression of NCR-sensitive genes in *C. parapsilosis*, similarly to *S. cerevisiae* and other fungi (55, 56). Genes involved in transport and in amino acid metabolism are upregulated.

We found that the function of several core transcriptional regulators of the NCR is conserved in *C. parapsilosis* and other fungi. *GAT1* and *GLN3* are well studied in *S. cerevisiae* and *C. albicans*, where they encode activators of NCR-sensitive genes (4, 12–14, 34–36, 46). Deletion of either of these genes in *C. albicans* results in growth defects on many nitrogen-limiting media (36), which we also observed with the equivalent deletions in *C. parapsilosis* (Fig. 1). Expression of *C. parapsilosis GAT1* was increased >60-fold during growth on isoleucine (see Data Set S1 in the supplemental material). Repression of *GAT1* expression during growth on preferred nitrogen sources in other fungi has also been reported (20, 46).

In *S. cerevisiae*, two transcriptional repressors (*GZF3* and *DAL80*) are required to maintain NCR as long as a preferred nitrogen source is available (1, 4, 6). There is only one ortholog (called *GZF3*) in *Candida* species, and that ortholog is poorly characterized. We recently reported that* GZF3* regulates biofilm formation in *C. parapsilosis*, although the underlying mechanism is unknown (47). Here, we demonstrate that expression of NCR-sensitive genes (*GAP2*, *MEP2*, and *GAT1*) is increased when *GZF3* is deleted in *C. parapsilosis*, showing that Gzf3 also mediates NCR in *Candida* species.

The function of several other specific nitrogen regulators is also conserved in *C. parapsilosis*. For example, *GCN4* is required to utilize most amino acids as the sole nitrogen source. Deletion of *PUT3* resulted in a growth defect when proline was the sole nitrogen source (Fig. 1). Put3 has recently been shown to regulate the ability of *C. albicans* to use proline as a nitrogen and carbon source, suggesting that its role as a nitrogen regulator is conserved across *Candida* and *Saccharomyces* species (57). However, the role of *DAL81* is distinctly different (Fig. 1).

Dal81 proteins have a GAL4-like zinc finger DNA binding domain, which is found only in fungi (58). They also contain a “middle homology region” that probably has a regulatory function (59). *DAL81* was first characterized in *S. cerevisiae* in the 1980s, when several studies showed that it was required to catabolize allantoin and urea (60–64). Talibi and Raymond (65) later discovered that Dal81, together with Uga3, binds to DNA sequences upstream of GABA-inducible genes. Uga3 and Dal81 are both required for normal GABA uptake and for expression of the GABA permease *UGA4* (66). Dal81 was subsequently shown to act synergistically with other transcriptional activators driving expression of metabolic genes. For example, Dal81 and Dal82 interact at the promoters of allantoin-responsive genes (67) and, in response to external amino acids, Dal81 facilitates binding of the transcriptional activators Stp1p and Stp2p at the promoters of *AGP1*, *BAP2*, and *BAP3* (31).

We found that GABA induction of gene expression in *C. parapsilosis* does not require *DAL81*, confirming that that its roles in *S. cerevisiae* and *C. parapsilosis* are very different. On the other hand, both *C. parapsilosis* and *C. albicans* require *UGA3* to utilize GABA as a nitrogen source (Fig. 1). We do not know if Uga3 acts alone in *Candida* species, or together with an as-yet-unknown activator, to control expression of GABA metabolism.

We used transcriptomic analysis to further characterize the function of *DAL81* in *C. parapsilosis*. During growth in rich media with complex nitrogen sources (YPD), deletion of *DAL81* resulted in a major upregulation of the arginine biosynthesis pathway, in at least two different genetic backgrounds. Figure 3 shows that expression of almost every gene involved in arginine transport and biosynthesis was increased when *DAL81* was absent, whereas expression of degradation enzymes was reduced. Furthermore, the expression levels of *ARG1* and *ARG3* were restored to wild-type levels when a single allele of *DAL81* was reintroduced into the genome. *DAL81* therefore regulates arginine metabolism in *C. parapsilosis*, both by repressing expression of arginine biosynthesis genes and by activating expression of arginine-degrading genes. The effect of deleting *DAL81* was not quite as dramatic when cells were grown in minimal (YNB) media with ammonium sulfate as the sole nitrogen source, though the expression level of arginine genes was still increased. Overall, expression of arginine synthesis genes was higher in YNB than in YPD, probably because there is no arginine present in the former media, which may mask the effect of deleting *DAL81* (Data Set S1).

In *C. albicans*, nitrogen starvation results in increased expression of *DAL81*, which, together with *STP2*, activates expression of the vacuolar transporter *AVT11* (20). Ramachandra et al. (20) generated an *in silico* model of the regulatory networks that govern nitrogen metabolism in *C. albicans* which predicts involvement of Dal81 in the mobilization of nitrogen from vacuoles during nitrogen starvation. When nitrogen-starved cells were fed with arginine, expression of both *DAL81* and *AVT11* was decreased, suggesting that nitrogen storage and mobilization are not important under these conditions (20). The model suggests that *DAL81* is an activator of *GAT1* when cells are fed with arginine and that it activates *STP1* and represses *GLN3* when cells are fed with bovine serum albumin; both arginine and bovine serum albumin are poor nitrogen sources. Our data show that *DAL81* represses, rather than activates, expression of arginine biosynthesis genes in *C. parapsilosis*. The roles of *DAL81* in *C. albicans* and *C. parapsilosis* may be different, though this remains to be experimentally tested. In addition, we do not know if Dal81 directly regulates arginine gene expression or if it acts through another transcription factor. It is, however, clear that Dal81 does not regulate GABA or allantoin metabolism in either *Candida* species.

We show that there has been a dramatic change in the targets of the Dal81 transcription factor between *S. cerevisiae* and *C. parapsilosis*. Transcriptional rewiring is not uncommon in fungi, and several studies have identified divergent transcriptional circuits in *S. cerevisiae* and *C. albicans* (68–71). Significantly, many of these rewiring events affect central metabolic pathways such as carbohydrate metabolism (71, 72), ribosome biogenesis (70), and nucleotide biosynthesis (68). Lavoie et al. (73) suggested that fungi repurpose core metabolic regulators in order to adapt to different environmental niches but that their function often remains within a similar metabolic “field.” This may also be true in *C. parapsilosis*, where Dal81 does not regulate nitrogen assimilation from GABA and allantoin but does regulate the expression of many genes associated with arginine metabolism.

## MATERIALS AND METHODS

### Media and strains.

All strains were cultivated in YPD broth (Formedium; catalog no. CCM0210) or on solid YPD agar (Formedium; catalog no. CCM0110) at 30°C. For phenotype screening, minimal media (0.19% yeast nitrogen base without amino acids and ammonium sulfate [Formedium; catalog no. CYN0501], 2% glucose, with or without 2% agar) was used as the base medium. For *S. cerevisiae* strains, 0.08 g/liter uracil was added. For SN152-derived *C. albicans* strains, 0.05 g/liter arginine was added. The medium was then supplemented as indicated. For drop test plates, overnight cultures were collected by centrifugation at 13,000 rpm at room temperature for 30 to 60 s. Cells were washed by resuspension twice in 1 ml phosphate-buffered saline (PBS), and centrifugation was performed each time as described above. Washed cells were resuspended in 1 ml PBS, diluted to an *A*_600_ of 0.0625 in 1 ml PBS, and divided into aliquots and placed at 200 µl per well in the wells of a 96-well plate. Strains were then serially diluted 1:5 in 200 µl PBS to reach a final *A*_600_ of 0.0001. A 2-µl volume of each dilution was then transferred to solid media using a 48- or 96-pin replicator. Plates were incubated at 30°C and photographed at 48 and 72 h.

*C. parapsilosis* gene deletion strains were constructed as described by Holland et al. (47). *S. cerevisiae* strains 23344c and SBCY17 (74) were kindly provided by Mariana Bermúdez Moretti (Table S1). The *C. albicans uga3Δ/Δ* deletion strain, and the SN152 parent strain (49), were provided from the laboratory of A. D. Johnson. *DAL81* was reintroduced into a *C. parapsilosis dal81* deletion strain as shown in Fig. S1. The *C. albicans dal81Δ* mutant strain was constructed using the CRISPR method described by Vyas et al. in 2015 (75). *C. albicans* STCA2 (Table S1) contains *CAS9* integrated at the *ENO1* locus. This was transformed with pV1090-DAL81, which encodes a single guide RNA (DAL81_Guide_B) directed against *DAL81* (Table S2) as well as nourseothricin resistance (NAT^r^) and integrates at the *RP10* locus. The plasmid was digested with KpnI/SacI and cotransformed with a repair template (CaDAL81-RTb; Table S2) designed to insert two premature stop codons after amino acid 199. Homozygous *DAL81* mutant strains were confirmed by PCR amplification using primers ChkB-Fw and Check_Rv and by sequencing a fragment amplified with primers SeqChk_Fw and Check_Rv. *DAL81* was deleted in *C. parapsilosis* 90-137 using a CRISPR-based method described by Lombardi et al. (54). A guide RNA targeting position (+354 bp) was introduced into plasmid pRIBO using primers CpDAL81_sgRNAa_T and CpDAL81_sgRNAa_B, generating plasmid pRIBO-DAL81a. A 5-μg volume of pRIBO-DAL81a was cotransformed with 8 to 10 μg of a linear repair template into *C. parapsilosis* 90-137. The repair template was generated by primer extension using 120-bp oligonucleotides containing 20-bp overlapping sequences (DAL81_RTdel_T and DAL81_RTdel_B) and consisted of 200 bp from 5′ and 3′ of the *DAL81* open reading frame (ORF), flanking a 20-bp unique tag. Transformants were selected by colony PCR using primers flanking the ORF (DAL81_UPST and DAL81_DWST), and PCR products were verified by Sanger sequencing. Homology-directed repair resulted in deletion of the entire *DAL81* ORF, replacing it with a 20-bp tag.

10.1128/mSphere.00028-18.4 TABLE S1  List of strains used. Download TABLE S1, DOCX file, 0.1 MB. Copyright © 2018 Turner et al. 2018 Turner et al. This content is distributed under the terms of the Creative Commons Attribution 4.0 International license.

10.1128/mSphere.00028-18.5 TABLE S2  List of primers. Download TABLE S2, DOCX file, 0.1 MB. Copyright © 2018 Turner et al. 2018 Turner et al. This content is distributed under the terms of the Creative Commons Attribution 4.0 International license.

### RNA isolation.

Overnight cultures were washed twice in PBS and diluted to a starting *A*_600_ of ~0.3 in the desired medium. Cultures were incubated at 30°C and 250 rpm for 4 to 5 h until an *A*_600_ of approximately 1.0 to 1.5 was reached. Cells were harvested by vacuum filtration through 0.45-µm-pore-size filters and washed by subjecting filters to vortex mixing in 5 ml PBS. Finally, cells were collected again by vacuum filtration and washed from filters using 500 µl RNAlater (Qiagen; catalog no. 76104). Cells were snap-frozen in liquid nitrogen and stored at −80°C. For most RNA sequencing (RNA-seq) experiments, RNA extractions were performed using a RiboPure RNA purification kit (yeast) (Ambion; catalog no. AM1926). RNA quality was assessed using an Agilent 2100 Bioanalyzer instrument and an RNA 6000 Nano kit (Agilent; catalog no. 5067-1511). For cDNA synthesis and for analysis of cells growing with isoleucine as the sole nitrogen source, RNA was isolated using a Bioline isolate II RNA minikit (catalog no. BIO-52072). RNA concentrations were determined using a Thermo Scientific NanoDrop 2000 instrument. All purified RNA was stored at −80°C.

### Quantitative real-time PCR.

PCRs were performed using two technical replicates and at least three biological replicates. Data from the technical replicates were averaged prior to statistical analysis. For cDNA synthesis, equal quantities of RNA (200 to 1,000 ng) from each sample were diluted in 4 µl nuclease-free distilled water (dH_2_O) and 1 µl 100 µg/ml oligo(dT)_15_ primer (Promega; catalog no. C1101) was added. Samples were incubated in a thermocycler at 70°C for 10 min and cooled to 4°C. Reverse transcription was performed using a Moloney murine leukemia virus (MMLV) reverse transcriptase kit (Promega; catalog no. M1701) with incubation at 37°C for 60 min and 95°C for 2 min. Each PCR mixture contained 1 µl cDNA, 7 µl dH_2_O, 2 µl 5 µM primer mix (Table S2), and 10 µl FastStart Universal SYBR green Master (Rox) (Roche; catalog no. 04 913 850 001). Reactions were performed in optical PCR tubes (Agilent; catalog no. 401427 and 401428) using an Agilent MX3005P QPCR system. Statistical analysis of biological replicates was carried out using the comparative threshold cycle (*C*_*T*_) method described by Applied Biosystems.

### RNA-seq.

RNA isolated from the YPD-grown *C. parapsilosis* CLIB214 and *C. parapsilosis dal81Δ* strains and from *C. parapsilosis* CLIB214 grown in YNB–2% glucose (with 10 mM isoleucine or 0.5% ammonium sulfate) was sequenced by BGI Global Genomics Services (100 bases; paired ends). RNA from *C. parapsilosis* CPRI and *C. parapsilosis dal81Δ* strains grown in YNB–2% glucose (with 10 mM GABA or 0.5% ammonium sulfate) was sequenced in-house. For in-house sequencing, RNA was quantified using a Qubit 2.0 Fluorometer (Life Technologies, Inc.). Library preparation was performed using an Illumina NeoPrep system and a TruSeq Stranded mRNA NeoPrep kit (Illumina; catalog no. NP-202-1001). The quality of the library was assessed using an Agilent 2200 TapeStation instrument with a high-sensitivity DNA 1000 kit (Agilent; catalog no. 5067-5584). Libraries were pooled and sequenced using an Illumina NextSeq sequencer with a NextSeq 500/550 High Output v2 kit (Illumina; catalog no. FC-404-2001) (75 cycles). All data were analyzed using established bioinformatic protocols (76). In brief, reads were mapped to the *C. parapsilosis* genome using TopHat version 2 (77), transcripts were counted using HTSeq (78), and differentially expressed genes were identified using DESeq2 (79). Genes with a log_2_FC value above 1 or below −1 and with an adjusted *P* value of <0.001 were retained.

### Accession number(s).

All data were submitted to the Gene Expression Omnibus databases under accession number GSE109034.
