# Supplementary figures and images for: Dal81 Regulates Expression of Arginine Metabolism Genes in Candida parapsilosis
Source: mSphere. 2018 Mar 7;3(2):e00028-18. doi: 10.1128/mSphere.00028-18 (PMC5853489; doi:10.1128/mSphere.00028-18)

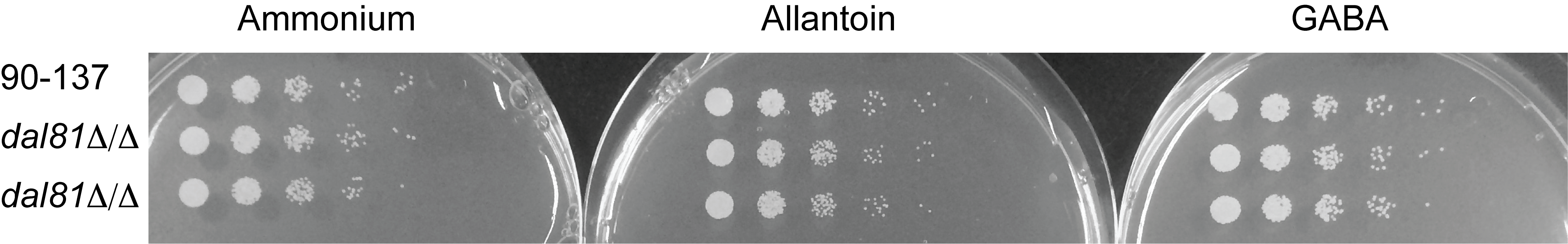

Supplement: FIG S2 [file sph001182486sf2.tif]
