# Supplementary material for: Dal81 Regulates Expression of Arginine Metabolism Genes in Candida parapsilosis
Source: mSphere. 2018 Mar 7;3(2):e00028-18. doi: 10.1128/mSphere.00028-18 (PMC5853489; doi:10.1128/mSphere.00028-18)
Supplement: TABLE S1 [file sph001182486st1.docx]

Table S1 List of strains used

| Strain | Description | Source |
| --- | --- | --- |
| *Candida parapsilosis* | |  |
| CLIB214 | Wild type strain | Type strain |
| CPRI | *leu2::FRT/leu2::FRT, his1::FRT/his1::FRT, frt::CmLEU2/frt::CdHIS1* | (1) |
| *gat1Δ/Δ* | *leu2::FRT/leu2::FRT, his1::FRT/his1::FRT, cpar2_500590::LEU2/ cpar2_5005901::HIS1* | (1) |
| *gzf3Δ/Δ* | *leu2::FRT/leu2::FRT, his1::FRT/his1::FRT, cpar2_800210::LEU2/ cpar2_800210::HIS1* | (1) |
| *gln3Δ/Δ* | *leu2::FRT/leu2::FRT, his1::FRT/his1::FRT, cpar2_101010::LEU2/ cpar2_101010::HIS1* | This study |
| *put3Δ/Δ* | *leu2::FRT/leu2::FRT, his1::FRT/his1::FRT, cpar2_208790::LEU2/ cpar2_208790 ::HIS1* | This study |
| *gcn4Δ/Δ* | *leu2::FRT/leu2::FRT, his1::FRT/his1::FRT, cpar2_806570::LEU2/ cpar2_806570::HIS1* | This study |
| *uga3Δ/Δ* | *leu2::FRT/leu2::FRT, his1::FRT/his1::FRT, cpar2_200790::LEU2/ cpar2_200790::HIS1* | This study |
| *dal81Δ/Δ* | *leu2::FRT/leu2::FRT, his1::FRT/his1::FRT, cpar2_800890::LEU2/ cpar2_800890::HIS1* | This study |
| *dal81Δ::DAL81* | *leu2::FRT/leu2::FRT, his1::FRT/his1::FRT, DAL81::LEU2/ cpar2_800890::HIS1* | This study |
| *90-137/ dal81Δ/Δ* | *dal81Δ::tag/ dal81Δ::tag* | This study |
| *Candida albicans* | |  |
| SN152 | *ura3/::imm434::URA3/ura3::imm434 iro1::IRO1/iro1::imm434 his1::hisG/his1::hisG leu2/leu2 arg4/arg4* | (2) |
| *uga3Δ/Δ* | *ura3/::imm434::URA3/ura3::imm434, iro1::IRO1/iro1::imm434, uga3::HIS1, uga3::LEU2, arg4/arg4* | (3) |
| STCA2 | *ENO1/eno1::CAS9* | (4) |
| *dal81*/** | *ENO1/eno1::CAS9, RP10::SAT1, dal81*/dal81** | This study |
|  |  |  |
| *Saccharomyces cerevisiae* | |  |
| 23344c | *MATα, ura3* | (5) |
| SBCY17 | *MATα, ura3, uga35Δ::natMX4* | (6) |

1. Holland LM, Schroder MS, Turner SA, Taff H, Andes D, Grozer Z, Gacser A, Ames L, Haynes K, Higgins DG, Butler G. 2014. Comparative phenotypic analysis of the major fungal pathogens *Candida parapsilosis* and *Candida albicans*. PLoS Pathog 10:e1004365.

2. Noble SM, Johnson AD. 2005. Strains and strategies for large-scale gene deletion studies of the diploid human fungal pathogen *Candida albicans*. Eukaryot Cell 4:298-309.

3. Homann OR, Dea J, Noble SM, Johnson AD. 2009. A phenotypic profile of the *Candida albicans* regulatory network. PLoS Genet 5:e1000783.

4. Vyas VK, Barrasa MI, Fink GR. 2015. A CRISPR system permits genetic engineering of essential genes and gene families. Sci Adv 1:e1500248.

5. Marini AM, Soussi-Boudekou S, Vissers S, Andre B. 1997. A family of ammonium transporters in *Saccharomyces cerevisiae*. Mol Cell Biol 17:4282-93.

6. Cardillo SB, Bermudez Moretti M, Correa Garcia S. 2010. Uga3 and Uga35/Dal81 transcription factors regulate UGA4 transcription in response to gamma-aminobutyric acid and leucine. Eukaryot Cell 9:1262-71.
