# Supplementary material for: Dal81 Regulates Expression of Arginine Metabolism Genes in Candida parapsilosis
Source: mSphere. 2018 Mar 7;3(2):e00028-18. doi: 10.1128/mSphere.00028-18 (PMC5853489; doi:10.1128/mSphere.00028-18)
Supplement: TABLE S2 [file sph001182486st2.docx]

Table S2 List of primers.

|  |  |  |  |  |  |
| --- | --- | --- | --- | --- | --- |
| Primers to generate gene deletion in *C. parapsilosis* | | | | | |
| Primer 2 | ccgctgctaggcgcgccgtgACCAGTGTGATGGATATCTGC (common to all) | | | | |
| Gene_ID | Primer 1 | Primer 3 | Primer 5 | Primer 4 | Primer 6 |
| CPAR2_101010 (*GLN3*) | TTACTCTATAGGAGTTAGGT | cacggcgcgcctagcagcggAAAAGAAATGGCCTGTGTGA | gcagggatgcggccgctgacACATCCTTGGCATGGGAGATagctcggatccactagtaacg | gtcagcggccgcatccctgcGAAAAAAGAGACAATAGGTT | TACATTAATCCTGGCATTTC |
| CPAR2_208790 (*PUT3*) | GTAAGTACATTATCTGTTCT | cacggcgcgcctagcagcggTGCTCTTTCAAGTGATTGAA | gcagggatgcggccgctgacGATATTGATCTCACCTGCCTagctcggatccactagtaacg | gtcagcggccgcatccctgcAATGAATAAGGGCTATACTT | ATAATACTAACTCCTTACTA |
| CPAR2_806570  (*GCN4*) | TTCATTCACCTTCCAGAGCC | cacggcgcgcctagcagcggTTTATTAAATCTTAGTGTAA | gcagggatgcggccgctgacCCGAGTTAGGAATGTTATGCagctcggatccactagtaacg | gtcagcggccgcatccctgcTTATTTAGATGCTTTTGTTA | ACTTCCCAAAAAGAGAAGAT |
| CPAR2_200790  (*UGA3*) | TACCTAGAAGTATTCACGGA | cacggcgcgcctagcagcggCAATTTGGACAAAAACTATC | gcagggatgcggccgctgacCGACTATTGTGACATACCTGagctcggatccactagtaacg | gtcagcggccgcatccctgcACAGTATATGATATATAGAT | CCCAAGTTTTTATCCGATAC |
| CPAR2_800890  (*DAL81*) | TCTGTCGTTGATGATTTGGT | cacggcgcgcctagcagcggTTGGAATTTAGTCTAAACCC | gcagggatgcggccgctgacCAGACTCTATCATGGTGTTGagctcggatccactagtaacg | gtcagcggccgcatccctgcATTAGAAGAATCTGTAACAC | CTCGTGTACATGATCCTGAA |
| Primers to check gene deletion in *C. parapsilosis* | | | | | |
| Gene_ID | 5' check-Fw | 3' check-Rv | ORF check-Rv | HIS1-check Rv/ HIS2-check Fw | LEU1-check Rv/ LEU1-check Fw |
| CPAR2_101010 (*GLN3*) | GCTCATTGTGTCAATTGTTT | TTCAGTTTCACCCTTCCAGG | TTTCCTTCTTCGTGGGCTCC | AAAATCAATGGGCATTCTCG/ TGGGAAGCAGACATTCAACA | GAAGTTGGTGACGCGATTGT/ GAAGTTGGTGACGCGATTGT |
| CPAR2_208790 (*PUT3*) | TATTCTTGTTCAGGAAACTT | AATCTCGCTTACAAACTGGT | GATGAGTCAAACGTGGGTGC | Same | Same |
| CPAR2_806570  (*GCN4*) | TCTTGCCCTAAACCCAAATC | TATCTCCAGATACGAGCTCG | GCCCTTGATCTTCTTGCTGC | Same | Same |
| CPAR2_200790  (*UGA3*) | TTGCCAAAAATCACAAAAGG | TGCAATAGCCTGGATAATAT | ATTGCCAGTTGTACTCCGCA | Same | Same |
| CPAR2_800890  (*DAL81*) | CTATTTGGGGGTTACTACTA | TAAAGTCGCCGGTAGTAGCT | TTGGTCTTTCGTCTTCGGCA | Same | Same |
| Primers for RT-PCR | | | | | |
| Gene_ID | Forward primer | | Reverse primer | |  |
| CPAR2_211040 (*UGA1*) | TCGCTTCGATTCCATTGGGT | | GTCGGTGACAATCTCGTCCA | |  |
| CPAR2_404090 (*UGA2*) | CCCCTCAGCAATGGGATCAA | | AACACCAGGTGGAAACCCTG | |  |
| CPAR2_804120 (*ARG1*) | AGAGCTGCATGCTACAAAGGA | | AGCTTGCACGGCAATGAATC | |  |
| CPAR2_601520 (*ARG3*) | AAGTTGGCTTGGGTTGGTGA | | TGTTGAGCAATCTGCCTTGC | |  |
| CPAR2_402870 (*MEP2*) | GGGCATTGCATGGAGTAGGT | | TTCATCCAGCCGCCATCAAT | |  |
| CPAR2_500590 (*GAT1*) | GCATCCTCCTCCTCCTCCTT | | TGGCGGTACATTCATGGGAC | |  |
| CPAR2_201570 (*ACT1*) | GAAGCTTTGTTCCGTCCAGC | | TGATGGAGCCAAAGCAGTGA | |  |
| Primers for CRISPR deletion of *DAL81* in *C. parapsilosis* | | | | | |
| CpDAL81_sgRNAa_T | TTCTTTCTTTTCCAAATTCAaacctgCTGATGAGTCCGTGAGGACGAAACGAGTAAGCTCGTC | | | | |
| CpDAL81_sgRNAa_B | GCTATTTCTAGCTCTAAAACTTGAGGATCGAGGTAACCTGGACGAGCTTACTCGTTTCGT | | | | |
| DAL81_RTdel_T | **caaacaggttcaactcagcataccttttcaacacgttagggacccgtcttactgcatccctgtcactgacacagcaactagggtttagactaaattccaa**CAGACTCTATCATGGTGTTG | | | | |
| DAL81_RTdel_B | **attgagaaatggagcccagtatcgtataacacaaggattcgcaaaaagtagtgtgttagagtttgttgatatacataatagtgttacagattcttctaat**CAACACCATGATAGAGTCTG | | | | |
| DAL81_UPST | gttatagtgtagagtaag | | | | |
| DAL81_DWST | GGAGGATGTTGACAAGGAG | | | | |
| Primers for complementing *DAL81* in *C. parapsilosis* | | | | | |
| Dal81_F_KpnI | GGAACTGGTACCTTTCACCACTTTGGATGTTG | | | | |
| Dal81_R_ApaI | GAATCTGGGCCCCCTGAACTCATCCTCAATCC | | | | |
| 5_Dal81_int_F | GTATCTTGTGGACTTGGGAC | | | | |
| 5_Dal81_int_R | CTGATTGTGATAGGTGTGATGG | | | | |
| 3_his1_int_F | GATCCACTAGTTCTAGAGCGG | | | | |
| 3_his1_int_R | TTCGTTGGAGAAGGTAATTGTG | | | | |
| Primers for CRISPR editing of *DAL81* in *C. albicans* | | | | | |
| DAL81_Guide_B | ACCTGATGTTGCTATTCGTG | | | | |
| CaDAL81-RTb-TOP | CAGTTTATTATCAAATGGATCAACTATTGTTCCACCTGATGTTGCTATTCGTG**agtaat**GACCT | | | | |
| CaDAL81-RTb-BOT | CAATAATGAATTATTCATGGCTGAATAATCTTGTATAGG TC**attact**CACGAATAGCAACAT | | | | |
| ChkB_Fw | CTGATCAAGCACCGAAATGA | | | | |
| SeqChk_Fw | CAATACTAATAATTGTGTCC | | | | |
| Check_Rv | TTGTATCAACATCATTCGAC | | | | |
